# Lipid Metabolism in Cartilage Development, Degeneration, and Regeneration

**DOI:** 10.3390/nu14193984

**Published:** 2022-09-25

**Authors:** Zhanpeng Su, Zhixian Zong, Jinxia Deng, Jianping Huang, Guihua Liu, Bo Wei, Liao Cui, Gang Li, Huan Zhong, Sien Lin

**Affiliations:** 1 Orthopaedic Center, Affiliated Hospital of Guangdong Medical University, Guangdong Medical University, Zhanjiang 524013, China; 2 Musculoskeletal Research Laboratory, Department of Orthopaedics & Traumatology, The Chinese University of Hong Kong, Prince of Wales Hospital, Hong Kong SAR, China; 3 Stem Cells and Regenerative Medicine Laboratory, Li Ka Shing Institute of Health Sciences, The Chinese University of Hong Kong, Prince of Wales Hospital, Hong Kong SAR, China; 4 Department of Prosthodontics, Yonsei University College of Dentistry, Seoul 03722, Korea; 5 Institute of Orthopaedics, Huizhou Municipal Central Hospital, Huizhou 516001, China; 6 Department of Pharmacology, Marine Biomedical Research Institute, Guangdong Key Laboratory for Research and Development of Natural Drugs, Guangdong Medical Unversity, Zhanjiang 524023, China

**Keywords:** lipid metabolism, cartilage, osteoarthritis, fatty acid, cholesterol, phospholipid

## Abstract

Lipids affect cartilage growth, injury, and regeneration in diverse ways. Diet and metabolism have become increasingly important as the prevalence of obesity has risen. Proper lipid supplementation in the diet contributes to the preservation of cartilage function, whereas excessive lipid buildup is detrimental to cartilage. Lipid metabolic pathways can generate proinflammatory substances that are crucial to the development and management of osteoarthritis (OA). Lipid metabolism is a complicated metabolic process involving several regulatory systems, and lipid metabolites influence different features of cartilage. In this review, we examine the current knowledge about cartilage growth, degeneration, and regeneration processes, as well as the most recent research on the significance of lipids and their metabolism in cartilage, including the extracellular matrix and chondrocytes. An in-depth examination of the involvement of lipid metabolism in cartilage metabolism will provide insight into cartilage metabolism and lead to the development of new treatment techniques for metabolic cartilage damage.

## 1. Introduction

Cartilage is a connective tissue dominated by the extracellular matrix (ECM) that lacks vascular, lymphatic, and nerve supply. Human cartilage is classified into three types: hyaline, fibrous, and elastic cartilage [1]. Among these, hyaline cartilage is the most common one in the body. With a glassy appearance and sitting on the surface of the joint, hyaline cartilage reduces the friction caused by joint movement. Two major components are present in the ECM of cartilage: collagens and proteoglycans. Proteoglycans are formed by glycosaminoglycans covalently linked to core proteins through side chains [2]. Hyaline cartilage contains a large amount of proteoglycans with a gel-like structure and water-rich glycosaminoglycan chains that provide compressive elasticity to the cartilage, which is the reason why cartilage can absorb and distribute the pressure applied to the joint [3]. Although they take up only 5–10% of the total volume of the cartilage, chondrocytes and their progenitor play an important role in maintaining the homeostasis of cartilage [4,5]. Chondrocytes synthesize the ECM and are embedded themselves in the matrix [6]. Chondrocytes and the ECM are functionally interrelated. Chondrocytes play a crucial role in cartilage formation, degradation and regeneration, and the ECM provides the microenvironment in which chondrocytes exist healthily and maintain the normal structure of cartilage [7]. Besides chondrocytes, progenitor cells are also identified in the cartilage which may be responsible for cartilage repair due to their self-renewal, multilineage differentiation, and migratory abilities [4].

Lipids including triacylglycerols, phospholipids, and cholesteryl esters play important roles in many physiological processes in the body [8]. They are known to act as energy storage substances and elemental components of the cell membranes, as well as precursors of inflammatory cytokines. Meanwhile, they also maintain metabolic homeostasis in association with intestinal flora, enzyme activators, glycosyl carriers, and regulating the function of immune cells [9,10,11]. The dysfunction of lipid metabolism, however, may lead to a number of disorders such as obesity, type II diabetes, and atherosclerosis [12]. Cartilage has many saturated fatty acids such as linoleic acid, oleic acid, and palmitic acid, which are involved in maintaining its structure and function. When lipid content or metabolism are disrupted, cartilage dysfunction results [13]. This article aims to review the function of lipid metabolism in the formation, degeneration, and regeneration of cartilage, which may shed light on potential therapeutics for cartilage illnesses.

## 2. Cartilage Development, Degeneration, and Regeneration

### 2.1. Chondrogenesis and Endochondral Ossification in Cartilage Development

**Figure 1:**
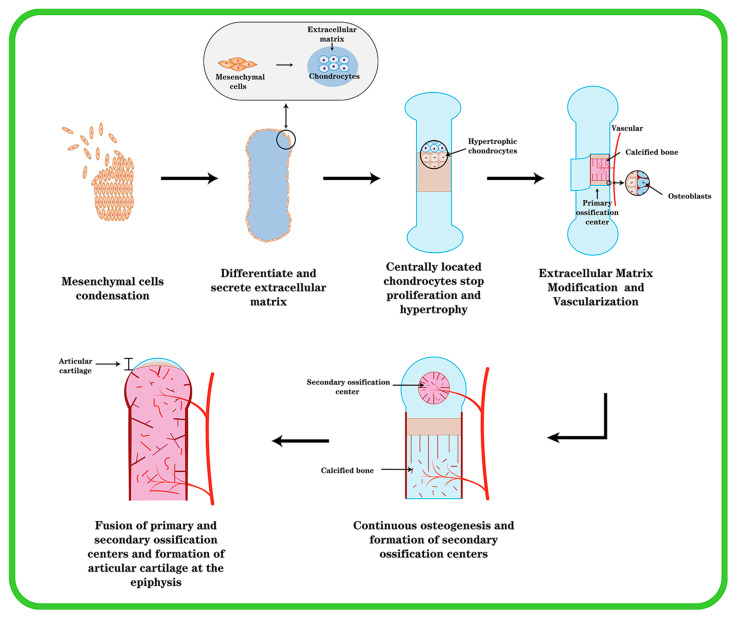
The formation of endochondral ossification and articular cartilage. Initially, mesenchymal cells undergo agglutination, mesenchymal cells in the center differentiate into chondrocytes and secrete type II collagen, chondrocytes continue to proliferate and secrete ECM, and cells around the agglutination express type I collagen and become cartilage membrane. The cartilage center’s cells then transform into hypertrophic chondrocytes, and blood vessels invade and create main ossification centers. Cartilage is progressively replaced by bone, and the cartilage membrane transforms into periosteum. At the ends of the elements, secondary ossification centers are created, and the epiphyseal cartilage plate is generated between the secondary and primary ossification centers. At last, the epiphyseal plate ossifies, leaving permanent articular cartilage at the element’s end.

Except for the cranium, the bones in the mesial and appendicular skeleton are generated from a hyaline cartilage template. The coagulation of mesenchymal cells, which occurs in the craniofacial region (e.g., middle ear bone and temporal bone) or in the mesoderm elsewhere in the body as the beginning of endochondral ossification, is essential for cartilage differentiation [14]. One of the earliest morphogenetic processes during embryonic development is the synthesis of cartilage, which serves as the starting point for osteogenesis inside cartilage, and then progresses to gradually forming bones and skeletal tissue. Mesenchymal cells condense, differentiate into chondrocytes, and then, cartilage tissue transforms into bone (Figure 1). This process is subject to stringent regulation by multiple stakeholders. The first stage is for undifferentiated mesenchymal cells to migrate to the osteogenesis site, where they begin to cluster together. The clumping of cells is induced by changed mitotic activity and the inability of cells to leave the center, which is caused by cell aggregation toward the center. This cell activity causes an increase in the density of mesenchymal stem cell (MSC) accumulation and a constant aggregation, but not an increase in cell proliferation.

This process could be mediated by the ECM promoting cell migration via intercellular adhesion molecules and gap junctions associated with increased intercellular contact, allowing for intercellular communication and small-molecule transfer. The process of mesenchymal cell condensation is critical in cartilage formation. This process is controlled by cell–cell and cell–matrix interactions, as well as the interrelationships of secreted substances with their corresponding receptors. [15]. Mesenchymal cells secrete an ECM rich in hyaluronic acid and type I collagen prior to coagulation, preventing intimate cell–cell interactions. Hyaluronan promotes cell motility. However, a rise in hyaluronidase activity abolishes cell–cell interactions induced by hyaluronan. The activation of one or more signal transduction pathways that results in the differentiation of mesenchymal cells into chondrocytes may entail cell–cell interactions. Cell–matrix interactions, in addition to cell–cell interactions, appear to be important in MSC coagulation. The condensation of mesenchymal cells generates a scaffold for the formation of skeletal elements within cartilage, determining the shape, size, location, and number of skeletal elements. 

Through the gradual differentiation of mesenchymal cells into chondrocytes and prechondral condensation, the ECM’s composition changes. Chondrocytes begin to produce cartilage-specific type II collagen, chondroitin sulfate-rich proteoglycans, aggrecan, and fibronectin, whereas type I collagen expression is suppressed [16]. Chondrocytes in the center of condensation undergo rapid proliferation and linear growth of skeletal elements, after which centrally located chondrocytes stop proliferating and enter the maturation process; after further differentiation and hypertrophy, chondrocytes express type X collagen, whereas type II collagen expression decreases, and cartilage development appears in a different zone [15]. Toward the end of the element are zones with a relatively flattened morphology where cells accumulate with each other and chondrocytes have a high proliferative capacity. Chondrocytes in the zone where proliferation is relatively quiescent (resting zone) are rounded. In the center of the element are chondrocytes that stop proliferating and become hypertrophic (the “hypertrophic zone”) [17]. The cells surrounding the condensate retain the fibroblast morphology and continue to express type I collagen, giving rise to the chondrocyte membrane. Chondrocytes undergo hypertrophy and cell death during the process of endochondral ossification, and hypertrophic chondrocytes play an important role in the process. There are two types of cartilage: temporary and permanent. The healthy cartilage in a joint is referred to as permanent or resting cartilage. Permanent cartilage has a low proliferative capacity and does not undergo terminal differentiation under normal conditions, whereas temporary cartilage will eventually form bone. A portion of chondrocytes undergo hypertrophic changes and transform into hypertrophic chondrocytes during their proliferation [18]. Cellular hypertrophy refers to the increase in cell size and volume. Both collagenase activity and the cellular matrix are altered during the process of chondrocyte hypertrophy. During hypertrophy, the ECM changes as a result of the chondrocyte volume change, resulting in tissue failure and an increase in collagen type X [19]. Hypertrophic chondrocytes express elevated levels of type X collagen, runt-associated transcription factor 2 (Runx2), and matrix metalloproteinase 13 (MMP-13). In contrast, cartilage markers such as aggregated glycan, type II collagen, and Sox9 were reduced in hypertrophic chondrocytes. Type X collagen is a well-known marker of chondrocyte hypertrophy. Although the function of type X collagen is unknown, its presence at sites of chondrocyte hypertrophy and calcification suggests that it may play a role in the early stages of bone formation within cartilage [20]. Most of these hypertrophic cells at the core of the element perish over time, whereas the ECM stays intact. In this region, hypertrophic cartilage produces MMP-13 to modify the extracellular matrix (ECM), thereby promoting vascular invasion and subsequent vascularization in a process that transforms cartilage tissue lacking vascular supply into blood-rich bone tissue and necessitates the initiation of angiogenic programs that require potent angiogenic factors such as vascular endothelial growth factor (VEGF) [21]. Osteoblasts penetrate cartilage via blood arteries and begin replacing it with mineralized bone. Endochondral osteogenesis [22] outlines the production of cartilage, hypertrophy, and mineralization, followed by bone formation. 

As stated, the process of endochondral osteogenesis takes place in growth plate cartilage, which differs from articular cartilage in terms of ECM content, cellular organization, and mechanical properties. Articular cartilage contains fewer chondrocytes and is embedded as separate cells in a distinct ECM with more collagen crosslinks than growth plate cartilage [21]. Mature articular cartilage has a banded organization that divides into a calcified layer, a deep layer, and a calcified layer from the articular cartilage surface toward the bone. The three groups of the non-hypertrophic Sox (Sox9/5/6) program are maintained in this cartilage, and the chondrocyte differentiation process is terminated to form permanent cartilage at the ends of the long bones [22].

### 2.2. Cartilage Homeostasis

The ECMs and chondrocytes have a mutually beneficial relationship: chondrocytes produce ECMs to form a highly organized molecular framework, and chondrocytes require the surrounding matrix to support their functions. Under normal physiological conditions, chondrocytes regulate the collapse and synthesis of the ECM in a dynamic balance. Chondrocytes are encapsulated in the ECM, and the stability of the ECM provides a stable microenvironment for chondrocytes. The mechanism of chondrocyte differentiation and phenotypic stability is regulated by a complex network of signaling molecules. When cartilage is injured, the chondrocyte phenotype changes, or even will become apoptotic, which is followed by ECM remodeling [23]. 

A wide range of signaling molecules influence cartilage matrix synthesis and maintenance, which are regulated via multiple signaling pathways. TGF-family members include BMPs, and are considered the major molecules in cartilage anabolism, along with insulin growth factor-1 (IGF-1), fibroblast growth factor-2 (FGF-2), and BMP-7. TGF-β binds to the type I receptors of activin-like kinases (ALK) ALK1 and ALK5, activating the Smad1/5/8 pathway and phosphorylating Smad2 and 3, respectively, causing a cascade of biological effects [24]. Smad2 and Smad3 inhibit chondrocyte hypertrophy as well as the entry of chondrocytes into the terminal differentiation state [25]. The activation of ALK5 and Smad2/3, on the other hand, induces chondrocyte synthesis of type II collagen and aggregated glycans, maintains chondrocyte stability, and plays an important role in chondrogenic differentiation regulation [26]. Reduced expression levels of ALK5 in senescent chondrocytes or OA cause disruption of the differentiation state of chondrocytes and promote the conversion of chondrocytes to a hypertrophic phenotype. In contrast, increased ALK1 expression levels activate the Smad1/5/8 signaling pathway, whereas Runx2 seems to have some synergistic effects with ALK1, promoting chondrocyte hypertrophy with increased synthesis of type X collagen and MMP-13, leading to cartilage matrix degradation [27].

BMP regulates cartilage development by regulating the Smad1/5/8 and Smad4 signaling pathways, and it is a critical regulator in all stages of cartilage development [28]. Various members of BMPs have different effects on chondrocytes, either in terms of cartilage matrix degradation and chondrocyte hypertrophy or in terms of chondrocyte proliferation and matrix synthesis. BMP can increase the synthesis of type II collagen and aggregated glycans in animal models of cartilage defects, whereas inhibiting BMP activity causes cartilage damage and reduced synthesis of proteoglycans [29]. In chondrocytes, BMP-7 is thought to induce extracellular matrix synthesis while inhibiting IL-1, IL-6, and fibronectin fragment-induced catabolism, promoting cartilage matrix synthesis while inhibiting catabolism [30]. BMP-2 and BMP-7 have different effects on chondrocytes, with BMP-2 promoting hypertrophy and BMP-7 inhibiting hypertrophy and maintaining cartilage stability [31].

### 2.3. Chondrocytes and Progenitor Cells in Cartilage Regeneration

Self-repair following cartilage injury is a tough procedure. When articular cartilage is injured, the mechanical loading of the cartilage changes, and oxidative stress mediates the harmful effects of shear stress on chondrocytes, resulting in chondrocyte senescence or death [32]. Normal articular cartilage is in a stable state of differentiation, secreting the ECM to preserve the structural integrity of the cartilage matrix and the function of the cartilage. Chondrocytes are induced to diversify into distinct phenotypes when OA arises as a result of changes in the chondrocyte microenvironment and cell death. Chondrocytes may undergo dedifferentiation alterations in OA, regaining the ability to proliferate, replenish chondrocytes that die in inflammation, or replace ECM-degrading components [33]. Both dedifferentiated chondrocytes and chondroprogenitor cells have the ability to proliferate. Indeed, numerous dedifferentiation marker genes, including collagen type II (IIA and IIB) and VI, tendin, and others, are increased in OA, implying a return of the chondroprogenitor phenotype [34]. Furthermore, during proliferation, osteoarthritic chondrocytes become senescent and hypertrophic, secreting proteins such as type X collagen and MMP-13 that are implicated in the matrix breakdown and calcification in OA [18].

Cartilage progenitor cells (CPCs), synovial-derived MSCs (SD-MSCs), synovial fluid-derived MSCs (SF-MSCs), bone marrow-derived MSCs (BM-MSCs), infrapatellar fat pad-derived MSCs (IF-MSCs), and MSCs from other origins all play a role in cartilage repair [35]. Bone marrow MSCs have been studied widely, and transplantation of bone marrow MSCs may reduce pain, enhance joint function, and halt the course of cartilage deterioration in OA patients [36]. Chondroprogenitor cells are found in both the superficial and deep zones of cartilage; however, the superficial zone seems to be more abundant, and the properties of chondroprogenitor cells vary by area. Chondroprogenitor cells in the deeper layers of cartilage tissue have a larger ability for chondrogenesis, are less phenotypically differentiated, have more flexibility, and may differentiate into a range of cell types. They can restore cartilage that has been damaged or degenerated. When cartilage is activated by injury or inflammation, the cells proliferate faster and migrate to the site of injury for repair. Superficial chondroprogenitor cells may originate from deeper layers, showing higher lubricant expression than the deeper counterpart, and proliferating slowly [37]. Quiescent MSCs are also discovered in the synovial membrane of the mouse knee, where they proliferate and differentiate to heal cartilage injury [38]. The majority of cells expressing growth and differentiation factor 5 (Gdf5) are located in the synovial lining, including fibroblast-like synoviocytes (FLS) that express lubricant (or proteoglycan 4 (Prg4)) and the Yes-associated protein (Yap) to maintain synovial lining proliferation and promote cartilage regeneration [39]. Besides, MSCs from the infrapatellar fat pad and synovial fluid may also play important roles in cartilage repair as well as OA development [40,41,42,43].

Cartilage damage generates an unfavorable microenvironment for chondrocytes and the ECM. As recruitment cues for bone marrow MSCs, cell lysates, ECM catabolic fragments, and high-mobility group box chromosomal protein 1 (HMGB1) can all function [18]. In damaged joints, growth factors, chemokines (chemokine (C-C motif) ligands (CCLs), chemokine (C-X-C motif) ligands (CXCLs), and chemokine (C-X3-C motif) ligands (CX3CLs)), and several other cytokines can function as recruitment signals to regulate endogenous bone marrow mesenchymal stem cells. Synovial fluid can create several chemokines when joint lesions develop, with CCL25, CXCL10, and XCL1 strongly attracting human subchondral mesenchymal progenitor cells, whereas the cell migration of other cells is missing or suppressed [44]. In response to recruitment cues, bone marrow mesenchymal stem cells may undertake directed motility and migration [45]. Bone marrow MSCs move into injured cartilage to produce a bone marrow MSC–chondrocyte co-culture system. Bone marrow MSCs can assist chondrocytes in regeneration, and chondrocytes can support bone marrow MSCs in differentiation and matrix synthesis, therefore interacting to heal cartilage [46]. In addition to regenerating the ECM, recruited bone marrow MSCs can ameliorate the microenvironment in damaged cartilage and support chondrocyte survival. MSCs can suppress the gene expression of COX2 (PTGS2), PGE2, TNF-α, IL-1β, IL-6, and CXCL8 (IL-8), as well as inflammatory proteins IL-6 and IL-8. CCL2, CCL3, CCL5, and CCL1 levels are also reduced [47]. In the presence of MSCs, the expression levels of chondrocyte fibrosis and hypertrophy genes were downregulated, whereas the expression levels of pro-chondrogenic-related genes, such as ACNA and COL2A1, were increased [47]. Mesenchymal stem cells can also have a supporting impact on chondrocytes by secreting paracrine cytokines such as PDGF and TGF-β, thus stimulating the proliferation of chondrocytes and playing an essential part in the repair of articular cartilage [48].

### 2.4. ECMs and Chondrocytes in Cartilage Degeneration

Cartilage is essential in joints, and its malfunction can lead to a variety of disorders, the most frequent of which is OA. OA is a widespread chronic degenerative disease characterized by cartilage degeneration, osteophyte formation, and joint sclerosis, with pain as the primary symptom. OA can affect any joint, including the joints of the hands and feet, hips, and knees [49]. OA is a considerable expense on society and families, as well as a serious global public health concern. The annual cost of OA treatment ranges from 1% to 5% of GDP, and with the rising trend of obesity and aging, the incidence and cost of OA will continue to increase [50].

Mechanical stress injury is a significant cause of OA. Injury induces the upregulation of Runx2 and IHH, as well as the expression of proteases. Aggrecan degradation is an early event in cartilage degradation, and lysosomal histone proteases found in cartilage can degrade glycoprotein at an acidic pH [51]. The aggrecan domain is cleaved at the interglobular structural domain (IGD) between the N-terminal G1 and G2 globular structural domains of aggrecan by ADAMTSs, which are zinc-dependent metalloproteases of the metalloprotein family. All of the ADAMTS enzymes degrade aggrecan, but ADAMTS-5 is the most active “aggrecanase” in vitro, followed by ADAMTS-4, and thus, both are thought to be the main enzymes responsible for the pathological cleavage of aggrecan on the Glu373~Ala374 bond in IGD [52]. Drugs that inhibit ADATMS-4/5 are currently being researched as a promising OA therapy that targets cartilage degradation and has the potential to reduce joint pain and restore normal function [53]. The specific order of degradation of cartilage tissue’s various components is unknown. Aggrecan is thought to protect against collagen degradation, which is a reversible process in OA. However, collagen degradation is irreversible, and cartilage cannot be repaired once collagen is degraded [54,55]. MMP-1, MMP-3, MMP-8, and MMP-13 are all involved in collagen degradation, and MMP-13 is considered to be the main collagenase with increased expression in OA cartilage [56,57]. In addition, other MMPs (e.g., MMP-28), a disintegrin and metalloproteinases (e.g., ADAM-12, ADAM-15), and ADAMTSs (e.g., ADAMTS-16, ADAMTS-17) are also increased in OA [58]. Cathepsin K, a papain-like cysteine protease, is the only enzyme other than collagenolytic MMP that hydrolyzes natural triple-helix type I and type II collagen. Cathepsin K expression in chondrocytes is increased in OA, and the enzyme is thought to be involved in collagen degradation in the cartilage matrix and subchondral bone [59].

Besides ECMs, chondrocytes also play an important role in cartilage health maintenance and disease development. The phenotype of chondrocytes is determined by the microenvironment in which they are located. Mechanical stress and pathological stimuli such as TNF-α, IL-1β, and NO cause chondrocytes to change into various phenotypes, and these phenotypes respond to inflammatory stimuli in various ways [60]. Chondrocytes of different phenotypes play different roles in OA, either repairing cartilage or degrading the joint, and among these phenotypes, the catabolic phenotype dominates, promoting cartilage matrix degradation [61]. The catabolic phenotype is founded on the secretion of various hydrolytic proteases. The anabolic phenotype is founded on ECM regeneration and repair (including type II collagen and proteoglycans). The hypertrophic phenotype is founded on type X collagen secretion followed by apoptosis. The dedifferentiated phenotype is based on type I collagen secretion. Additionally, the chondrogenic phenotype is dominated by synthetic fetal type II collagen (IIA) and type III collagen [62].

## 3. Lipid Metabolism in Cartilage

Metabolic disorders such as obesity, diabetes mellitus, and cardiovascular diseases have been identified as risk factors of OA, and they are regarded as predictive of faster deterioration of the OA joint. Emerging evidence reveals the role of common pathways of metabolic diseases and OA, such as lipid metabolism, may have a direct systemic effect on joints [63]. 

**Figure 2:**
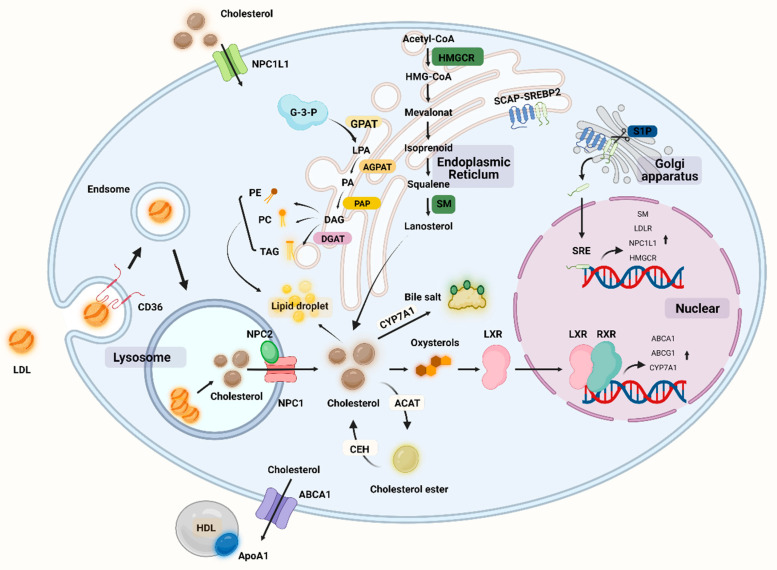
Overview of lipid metabolism in cells including chondrocytes. NPC1L1: Niemann–Pick C1-like 1, SM: squalene monooxygenase, HMGCR: hydroxymethylglutarate monoacyl coenzyme A reductase, HMG-CoA: 3-hydroxy-3-methylglutaryl-coenzyme A, CEH: cholesteryl ester hydrolase, ACAT: acyl coenzyme cholesterol acyltransferase, CYP7A1: cholesterol 7α-hydroxylase, LXR: liver x receptor, RXR: retinoic acid X receptor, SCAP: SREBP cleavage activation protein, SREBP: sterol-regulatory element binding protein, TAG: triglyceride, PE: phosphatidylethanolamine, PC: phosphatidyl choline (lecithin), GPAT: glycerol-3-phosphate acyltransferase, AGPAT: lysophospholipid acyltransferase antibody, DGAT: diacylglycerol acyltransferase, G-3-P: glyceraldehyde 3-phosphate, LPA: lysophosphatidic acid, PA: phosphatidic acid DAG: glycerol diester, PAP: phosphatidic acid phosphatase. (Figure was created with Biorender.com and accessed 20 September 2022).

Lipids are large molecules with a multitude of complex structures that play an important role in maintaining cellular functions [64]. There are four main lipid classes in the human body, including triglycerides (TGs), fatty acids (FAs), cholesterol, and phospholipids. Fats are important nutrients in the daily diet because they provide energy but also serve a variety of other functions, such as aiding in the absorption of fat-soluble vitamins and playing a role in the production and regulation of eicosanoids [65]. Fats are digested by lipase into fatty acids, glycerol, and monoacylglycerols, which are subsequently absorbed by the small intestine via bile salts [66]. Fatty acids can be absorbed by cells and become an important component of cell membranes. Lipids and their metabolic components can play an important role in signal transduction as second messengers between cells, and lipids are important in cartilage (Figure 2) [67].

In cartilage, the transport of lipids is a complex process. The complex structure of the cartilage matrix endows articular cartilage the ability to resist compressive loads and also determines how molecules diffuse into the matrix [68]. Recent research suggests two modes of lipid transport: synovial fluid diffusion and subchondral bone exchange. Synovial fluid is thought to be the primary source of molecules in articular cartilage metabolism. It has been discovered that synovial fluid, not subchondral bone, provides sufficient nutrients to maintain the structure and function of mature articular cartilage. The diffusion of solute molecules in the synovial fluid is significantly impacted by compression and circulatory loads in the joint [69]. The cartilage matrix is negatively charged, and only small, neutral molecules can diffuse freely through the joint fluid, whereas the diffusion of large and small negatively charged molecules is restricted [68]. To increase solubility, lipids are typically present in the blood as lipoproteins or bound to carrier plasma proteins. Once bound to large molecules, the transport of lipids into cartilage will be restricted. Since size is the determining factor for molecular diffusion, for the diffusion of non-charged small molecules, there is no discernible difference between immature and mature cartilage [70]. 

In contrast to synovial fluid, vascular penetration from the subchondral bone allows molecules to be transported in mature joints’ calcified cartilage. The bone–chondral junction in articular cartilage is viewed as a complex three-dimensional structure known as the tidemark, which enables the transport of molecules as the uncalcified cartilage extends across the calcified cartilage and makes contact with the vessels of bone and bone marrow [71]. However, in mature joints, there is calcified cartilage at the bone–chondral interface, and this natural barrier greatly limits the entry of lipids and other nutrients from calcified cartilage to non-calcified cartilage [72]. Recently, it was reported that regardless of body mass index (BMI), abnormal lipid accumulation in OA chondrocytes may be responsible for the development and progression of OA [73].

### 3.1. Fatty Acid

Previous evidence shows that total free fatty acids (FFAs) and the accumulation of substantial lipids in articular cartilage have been reported in OA patients prior to the appearance of histopathological OA features [74]. A higher level of saturated fatty acids (SFAs) is also closely related to the progression of cartilage degradation, and SFA deposition in cartilage may alter its metabolism and lead to cartilage damage [73]. Even an SFA-rich diet is associated with increased OA-like pathophysiological changes [75]. 

**Figure 3:**
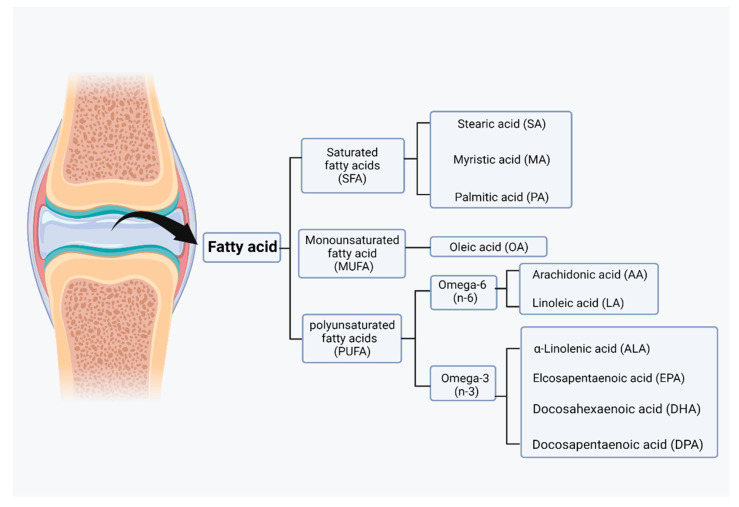
Classification of fatty acids in joint. Based on the degree of saturation, fatty acids can be categorized as saturated fatty acids (SFAs), monounsaturated fatty acids (MUFAs), or polyunsaturated fatty acid (PUFAs). Those fatty acids can be further categorized into lower grades. (Figure was created with Biorender.com and accessed 20 September 2022).

Fatty acids can be taken up from food, but they can also be synthesized by acetyl coenzyme A through a series of reactions in glycolysis and the citric acid cycle for lipid synthesis. However, acetyl coenzyme A itself is a byproduct of fatty acid oxidation. Acetyl coenzyme A, malonyl coenzyme A, and NADPH are required for the synthesis of fatty acids by fatty acid synthase (FAS) [76]. Malonyl coenzyme A is an important inhibitor of the carnitine/palmitoyl shuttle system and a C2 donor in the de novo synthesis of fatty acids. Two different enzymes, acetyl coenzyme A carboxylase 1 (ACC1) and acetyl coenzyme A carboxylase 2 (ACC2), have evolved for fatty acid synthesis and oxidation [77]. Based on their carbon chain length, fatty acids can be classified into short-chain fatty acids (SCFAs), medium-chain fatty acids (MCFAs), and long-chain fatty acids (LCFAs). Based on their degree of saturation, fatty acids can be categorized as SFAs, monounsaturated fatty acids (MUFAs), or polyunsaturated fatty acids (PUFAs), and each fatty acid has different biological effects (Figure 3) [78].

Stearate, a proinflammatory fatty acid, can partially activate hypoxia-inducible factor 1 (HIF-1) in chondrocytes to induce inflammation [79]. Palmitate acts as another proinflammatory fatty acid and catabolic factor in OA, which induces cystathione activation and cell death, stimulates normal chondrocytes via IL-1, and upregulates IL-6 and cyclooxygenase 2 (COX2) expression in chondrocytes through the Toll-like receptor 4 (TLR-4) signaling pathway [80]. Through the NADPH oxidase 4 (NOX-4) signaling pathway, high palmitic and oleic acid concentrations in articular cartilage mediate ROS production and chondrocyte apoptosis [81]. 

Differences in the desaturation sites of unsaturated fatty acids have resulted in the emergence of two distinct families of essential fatty acids: omega-3 and omega-6, which play different roles in the body. Alpha-linolenic acid (ALA) and linoleic acid (LA) are the dietary precursors of omega-3 unsaturated fatty acids (n-3 PUFAs) and omega-6 unsaturated fatty acids (n-6 PUFAs). In alpha-linolenic acid (ALA), 18 carbons and 3 double bonds are present, whereas 18 carbons and 2 double bonds are present in linoleic acid (LA) (18:2). Alpha-linolenic acid (ALA) and linoleic acid (LA) are both essential fatty acids because linoleic acid is desaturated by D15 desaturase to produce alpha-linolenic acid (ALA), but humans lack D15 desaturase. The conversion of n-3 unsaturated fatty acids to eicosapentaenoic acid (EPA FA, 20:5, n-3) and docosahexaenoic acid is possible (DHA FA, 22:6, n-3). In inflammation, omega-6 unsaturated fatty acids such as linolenic acid (LA FA, 18:2, n-6) are converted to gamma-LA (GLA FA, 18:3, n-6) and arachidonic acid (AA FA, 20:4, n-6) and play their own unique roles [82]. These fatty acids are precursors to a wide variety of bioactive lipids. In contrast to the proinflammatory effects of SFAs and n-6 PUFAs, omega-3 PUFAs can be oxidized to specialized proresolving molecules (SPMs), such as resolvins, protectins, and maresins, as well as other oxylipins with potent anti-inflammatory and pro-catabolic functions. SPMs function as signaling molecules that promote the restoration of vascular integrity, tissue regeneration, and repair, and decrease inflammation by inhibiting inflammatory lipid mediators and cytokines [83]. n-6 PUFAs, like SFAs, have proinflammatory effects that are mediated by lipid metabolites produced downstream of the precursor. Arachidonic acid is found in n-6 PUFAs and can be converted by cyclooxygenase (COX) and lipoxygenase (LOX) to eicosanoid compounds, which are biologically active lipids such as prostaglandins (PGs), thromboxanes (TXs), leukotrienes (LTs), and hydroxyeicosatetraenoic acids (HETEs). Due to the requirement for the same enzyme class for the conversion of a-linolenic acid to EPA and arachidonic acid to arachidonic acid, the synthesis of EPA and arachidonic acid will compete to regulate anti-inflammatory and proinflammatory homeostasis [84]. Normal cartilage typically contains low levels of n-6 polyunsaturated fatty acids (PUFAs) and high levels of n-9 fatty acids. Because the n-9 fatty acids may not function as a substrate for cyclooxygenase (COX), the cartilage is protected from prostaglandin (PG)-induced inflammatory responses. During aging, n-9 PUFAs in cartilage decrease while n-6 PUFAs increase [85]. Arachidonic acid is a widely distributed n-6 unsaturated fatty acid with 20 carbon and 4 double bonds (20:4) that is present in cell membranes and is an essential component of the lipid bilayer. However, in the presenting of inflammatory factors, phospholipase digestion of lipid molecules is stimulated, allowing arachidonic acid to be liberated in the cell membrane and then converted to PGH2 by COX, and then to leukotrienes by lipid oxidase. In diseased articular cartilage, the prostaglandins and leukotrienes produced by n-6 PUFAs were found to have strong proinflammatory and catabolic effects [86]. Furthermore, levels of prostaglandin E2 (PGE2) and 15-HETE (an oxytocin derived from arachidonic acid) in the synovial fluid of patients with OA were found to correlate with the degree of lesions [87]. 

Prostaglandin analogues are synthesized from PGH2 by activating PGD synthase or PGE synthase (PGEs). There are at least four types of PGE2 receptors (EP) in humans (EP1, EP2, EP3, and EP4), and different PGE2 receptors (EP) on the chondrocyte surface bind to PGE2 with different effects. When EP2 and EP4 are stimulated simultaneously in rats, for instance, they promote chondrocyte differentiation [88]. In the diseased joints of OA patients, synovial fibroblasts and chondrocytes produce proinflammatory cytokines (e.g., IL-1β and TNF-α), which result in the upregulation of the expression of MMPs and cause degenerative changes in articular cartilage. Additionally, that research reveals that PEG2 can inhibit the expression of MMP-1 and MMP-13 via EP4 and inhibition of the MKK4-JNK MAP kinase-c-JUN pathway [89]. IL-1β stimulates the production of PGE2 and upregulates the expression of EP1 and EP4 in rabbit chondrocytes, thereby increasing the sensitivity of chondrocytes to prostaglandins; however, the biological effects induced by the subsequent binding of the receptor to PGE2 are dependent on the cell’s state [90]. Microsomal prostaglandin E synthase 1 (mPGES-1), the terminal enzyme of PGE2 synthesis, is upregulated in many inflammatory conditions, including OA, and is regulated by MAP kinase phosphatase 1 (MKP-1), an enzyme that limits the activity of the proinflammatory MAP kinases p38 and JNK; thus, MKP-1 may be a new target for the treatment of OA [91]. The response of chondrocytes to different levels of PGE2 varies according to COX/mPGES and EP expression levels, mechanical stress stimulation in the diseased joint, and the action of growth factors and cytokines.

### 3.2. Cholesterol Metabolism

Total cholesterol is derived from the biosynthetic pathway of cellular cholesterol and dietary intake. Most tissues in the body, including chondrocytes, can synthesize cholesterol, and the liver is the primary site of synthesis, which occurs in the endoplasmic reticulum and cytoplasm of cells [92]. The synthetic enzymes required for cholesterol synthesis are found in the cytoplasm and cytosolic reticulum and are produced through a series of enzymatic reactions in which ATP provides energy and NADPH provides hydrogen. Cholesterol synthesis begins with acetyl coenzyme A, from which 3-hydroxy-3-methylglutaryl coenzyme A (HMG-CoA) is synthesized, followed by mevalonate (MVA), isopentenyl pyrophosphate (IPP), and finally, squalene is converted to cholesterol [93]. Cholesterol is important in the human body not only as a structural component of cell membranes, but also as a precursor to biosynthetic compounds such as bile acids, vitamin D, and some steroid hormones. Cholesterol is converted in the adrenal cortex to adrenocortical hormones, in the gonads to sex hormones such as androgens, estrogens, and progestins, in the skin to 7-dehydrocholesterol, by ultraviolet radiation to vitamin D3, and in the liver to bile acids, which aid in lipid digestion and absorption [94,95]. Along with other intermediates of the cholesterol biosynthesis pathway, cholesterol regulates signaling molecules including Ras, Hedgehog, and Rho [96,97,98]. 

Given that cholesterol is a major component of cell membranes, any alteration may impair the function and fluidity of cell membranes, resulting in abnormal cellular behavior and cholesterol accumulation [93]. Cholesterol is distributed in chondrocytes as scattered dots, primarily in the nucleus. Cholesterol is important in promoting chondrocyte differentiation and maturation. It was discovered in animal experiments. Cholesterol may regulate cartilage formation and promote endochondral osteogenesis by altering the activity of IHH, which is inhibited when cholesterol synthesis is inhibited [99]. However, inflammatory processes can be triggered by the accumulation of persistent low-density lipoproteins (LDLs) in the cartilage extracellular matrix. The LDL is the primary carrier of cholesterol from the liver to the cells, and once taken up by the cells via the LDL receptor (LDLR), lysosomes hydrolyze LDL cholesterol to release cholesterol [100]. Oxidized LDLs (ox-LDLs) binding to the chondrocyte LOX-1 stimulates ROS production in chondrocytes and activates NF-κB, thereby promoting the expression of catabolic genes such as MMP-1, MMP-9, ADAMTS-5, and ADAMTS-13 [101,102,103]. In addition, it has been discovered that ox-LDLs binding to LOX-1 under conditions of oxidative stress promotes the expression of the chondrocyte runt-related transcriptional gene 2, which induces chondrocyte hypertrophy and the production of type X collagen [104]. C-reactive protein (CRP) is a sensitive indicator of inflammation, and OA is associated with increased levels of CRP and interleukin-6 (IL-6) [105]. Recent studies clearly demonstrate the link between inflammation and imbalance in cholesterol homeostasis, with osteoarthritic cartilage exhibiting a considerable downregulation of cholesterol efflux gene (e.g., ApoA1 and ABCA1) expression as compared to normal cartilage. When cholesterol accumulates in cells, the liver X receptor (LXR) transcription factor is activated and binds to the promoter sequence of the ATP-binding cassette transporter protein A1 (ABCA1) gene, and ABCA1 plays a key role in cholesterol efflux by acting as a lipid pump to efflux intracellular cholesterol and phospholipids to apolipoprotein A1 (ApoA1), a major protein component of HDLs [106]. ABCA1 appears to have a function in anti-inflammation; ABCA1 controls the production of inflammatory proteins and chemokines in macrophage cells, and an increase in intracellular free cholesterol content in ABCA1 gene-deficient macrophages is associated with an increase in the macrophage inflammatory response [107]. Cholesterol metabolism-related proteins have a rigorous regulation mechanism in chondrocytes, with ApoA1 and ABCA1 expression increasing during differentiation and decreasing during hypertrophic differentiation. Serum amyloid A (SAA) has been reported to have proinflammatory effects, and SAA levels in OA plasma and synovial fluid rise with an increasing Kellgren–Lawrence grade in OA patients [108]. ApoA1 binds and transports cholesterol during chondrocyte differentiation, but it is possible that ApoA1 is replaced by SAA in response to inflammatory factor stimulation and is present in inflamed hypertrophic chondrocytes, as the mRNA for SAA is found to be maximally expressed when chondrocytes undergo hypertrophy [109]. ApoA1 stimulates the production of IL-6, MMP-1, and MMP-3 in primary chondrocytes and fibroblast-like synoviocytes, and increased SAA, HDL, and LDL concentrations lead to the inflammatory features of the cholesterol regulation-related protein ApoA1 in chondrocytes [110]. All protein regulation inside chondrocytes aims to strictly manage cholesterol levels in order to prevent hypercholesterolemia from depositing cholesterol in the joint. High cholesterol levels may play a role in OA etiology. We are familiar with hypercholesterolemia, which causes atherosclerosis of the blood arteries, damaging them and lowering the blood supply. When there is an accumulation of cholesterol in the joints, there is a danger of vascular injury, which impairs the blood flow to the subchondral bone. When cartilage loses oxygen and nutrients, this may result in histopathology and contribute to the development of OA [111]. Furthermore, hypercholesterolemia may also cause lipid oxidation and deposition in tissues, resulting in cartilage damage. In the presence of excessive cholesterol intake, the development of medial joint OA is significantly correlated with cholesterol exposure. In mice fed a cholesterol-rich diet and devoid of the low-density lipoprotein receptor (LDLr-/-), the size of the osteophyte at the edge of the tibial plateau increased [112]. The increased expression of cholesterol hydroxylases (CH25H and CYP7B1) and oxysterol metabolite synthesis, increased cholesterol absorption and cholesterol content, and increased expression of retinoic acid-related orphan receptor alpha (ROR) encouraged chondrocyte hypertrophy and catabolic-related proteins (e.g., MMP-13, ADAMTS-4, and ADAMTS-5). The severity of OA was reduced in mice whose cholesterol levels were lowered [113]. The process of cholesterol-related synthesis and regulation can be seen in Figure 2.

Cholesterol can also function as a signaling agent in chondrocytes, and signaling during cholesterol overload can increase LXR, upregulate genes for cholesterol efflux-related proteins (such as ABCA1 and ApoA1) to prevent cholesterol accumulation in cells, and limit chondrocyte hypertrophy [114]. SREBP transcription factors are crucial processes for regulating cholesterol homeostasis, and there are three SREBP proteins, SREBP-1a, SREBP-1c, and SREBP-2, each of which has a particular role. SREBP-2 is involved in activating cholesterol-related genes, boosting cholesterol absorption and synthesis, interacting with Smad3, and engaging in the OA process [115]. Through the TGF-β1/Akt/SREBP-2 pathway and the cholesterol efflux-related genes ABCA1 and ApoA1, miR-33a controls cholesterol production in OA chondrocytes. The expression of miR-33a and its host gene SREBP-2 was dramatically increased in OA chondrocytes, and cholesterol production via the TGF-1/Akt/SREBP-2 pathway stimulated a significant increase in chondrocyte MMP-13 expression levels; hence, the OA phenotype is enhanced and contributes to the inflammatory process [116]. Cholesterol can potentially facilitate as a second messenger in the signaling process. Hedgehog signaling is critical in the development of bone and cartilage. The G-protein-coupled receptor-like protein Smoothened (SMO) and downstream effectors transduce Hedgehog signaling pathway activation, and cholesterol is an essential component of the HH signaling pathway. Both cholesterol and sterol can activate HH signaling and cause SMO, but sterol deficiency is sufficient to inhibit HH signaling and cholesterol is required for HH activation. Subchondral bone has been demonstrated to stimulate HH signaling in animal OA models and to decrease the course of the lesion in vivo by SMO blocking [117]. Upregulation of the enzymes human 3-hydroxy-3-methylglutaryl coenzyme A synthase 1 (HMGCS1) and cytochrome P450 oxidase (CYP51A1), which synthesize cholesterol in chondrocytes, increases cholesterol biosynthesis during chondrogenesis, and cholesterol synthesis can promote the formation of growth plate cartilage by protecting against apoptosis and IHH expression [99,118].

### 3.3. Phospholipid Metabolism

Except for mature red blood cells, phospholipids can be synthesized in all human tissues, with the liver and kidneys serving as the primary sites. Phospholipids can be classified as either glycerophospholipids or sphingolipids, which are composed of glycerol and sphingomyelin, respectively. Sphingolipids are primarily synthesized in brain tissue. Coenzymes such as pyridoxal phosphate, NADPH+, and H+ are required in the reaction process, and the basic raw materials are palmitoyl coenzyme A and serine. Sphingolipids are formed after the formation of sphingomyelin and ceramides. Sphingomyelinase, an enzyme that hydrolyzes the phosphate bond to produce phosphorylated choline and ceramides, affects the degradation and metabolism of sphingolipids. Glycerophospholipids are the most common type of phospholipid. After being synthesized in the endoplasmic reticulum of the cytoplasm and processed by the Golgi apparatus, glycerophospholipids can be used to form cell membranes or secreted as lipoproteins outside the cell. Cardiolipin (CL) is a characteristic phospholipid of the inner mitochondrial membrane, where its synthesis also occurs. In addition to constituting biological membranes, glycerophospholipids are also bioactive components of the bile and membrane surface. Glycerophospholipids are also essential for protein recognition and cell membrane signaling [119,120]. 

LPCAT and PLA are critical for the metabolism of glycerophospholipids since they enhance the conversion of PC to lysolecithin (LysoPC). Elevated amounts of LPCAT4 and the essential regulating enzyme phospholipase A2 (PLA2) and its isoenzyme PLA2G5 have been discovered in OA cartilage, leading to increased levels of LysoPC. LysoPC is not only linked to an increased incidence of OA, but it may also be utilized to clinically predict arthroplasty after 10 years. In clinical investigations, the ratio of LysoPC to PC in synovial fluid has been identified as a novel marker for OA. LysoPC is a potent membrane lysis agent, and elevated levels are linked to chondrocyte apoptosis and cartilage degradation in OA, as well as to promoting cartilage inflammation by producing inflammatory lipid factors throughout the transition process [121]. In addition to its function in chondrocyte differentiation, hypertrophy, and mineralization, LPCAT4 is also involved in chondrocyte hypertrophy and mineralization. In mice, LPCAT4 mRNA expression was elevated in the hypertrophic zone, and LPCAT4 mRNA expression was also elevated in ATDC5 cells at late stages of development. When LPCAT4 was inhibited, the mRNA levels of cartilage differentiation markers, including Col10, alkaline phosphatase (ALP), aggregation protein (aggrecan), and Col10, were decreased [122]. OA synovial fluid is exposed to several proinflammatory stimuli that encourage the elevation of phospholipase A2 expression, and an increase in phospholipid composition may influence the inflammatory state of the joint [122]. Ceramide is one of the substrates for sphingolipid synthesis; it may be digested by ceramidase to generate sphingosine, which can then be phosphorylated by the sphingosine kinase to generate sphingosine-1-phosphate (S1P) [123]. Explants of rabbit articular cartilage treated with high concentrations of C2-ceramide elevated the matrix metalloproteinase-stimulated MMP-1, -3, and -13 expression and triggered chondrocyte death [67]. S1P stimulates chondrocyte proliferation by inducing phospholipase c-mediated intracellular calcium release while also activating the extracellular signal-regulated kinase (ERK) and p38 mitogen-activated kinase [124].

## 4. Interaction of Lipid Metabolism with Other Metabolisms 

Vitamin D (VitD) is a micronutrient that may be taken through diet, but is mostly created in the human body through the utilization of UV-provided energy. When exposed to UV light, the process of cholesterol production creates 7-deoxycholesterol (vitamin D prodigiosa), which transforms into provitamin D3. Provitamin D3 is converted to 25(OH)D3 in the liver by 25-hydroxylases (CYP2R1 and CYP27A1). 25(OH)D-1-hydroxylase (CYP27B1) transforms 25(OH)D3 to 1,25(OH)2D3. VitD circulates in the body and reaches tissues with vitamin D receptors (VDRs), including bone and chondrocytes. Most biological effects of VitD are mediated by VDRs, which form heterodimers with retinoid X receptors and regulate target genes containing VDR response elements (VDREs), increasing calcium absorption and bone formation. VitD affects calcium and phosphorus homeostasis and promotes calcium absorption and appropriate bone mineralization [125]. It is intimately connected to lipid metabolism, and VDR activity governs the process. As a key enzyme in the synthetic cholesterol pathway, DHCR7 is controlled by the sterol regulatory element binding protein (SREBP), together with the other enzymes involved in cholesterol production. The proteasomal degradation of DHCR7 decreases cholesterol synthesis, hence, changing the flow to vitamin D3. DHCR7 regulates the synthesis of vitamin D and cholesterol [126]. SREBP regulates lipid homeostasis by binding to and anchoring in the endoplasmic reticulum with the SREBP cleavage activator protein (SCAP), which is needed for SREBP activation (Figure 2). It has been discovered that 25-hydroxyvitamin D (25OHD) suppresses SREBP activation independently of VDRs, affecting SREBP activation via the initiating protein hydrolysis processes and ubiquitin-mediated SCAP degradation. 25OHD physically binds to SCAP at first, allowing serine protease to hydrolyze the SREBP-binding domain in SCAP. Then, the SREBP-binding domain is hydrolyzed, enabling 25OHD-dependent ubiquitination. SREBP is destabilized as a result of SCAP deterioration. As a result, downstream adipogenesis-related genes are downregulated in response to SREBP [127]. The activation of the VDRs by vitamin D increases the expression of MOGAT1 and LPGAT1 in the MG pathway and AGPAT2 in the glycerol-3-phosphate pathway. The activation of VDRs also causes an increase in phosphatidate cytidylyltransferase 1 (CDS1) expression, which facilitates the conversion of PA to CDP-DAG, PI, and PG [128]. VitD has a vital regulatory function in bone and cartilage, and VitD deficiency can result in normal structural alterations in patellar tendons and collateral ligaments due to fatty infiltration and inflammation. Low VitD levels are linked to muscle weakness and poor function, and muscle injury leads to diminished joint cushioning capacity, resulting in cartilage deterioration, which is considered to be connected with early OA. VitD supplementation lowers fatty infiltration and inflammation while also benefitting muscular function. Leptin induces cartilage deterioration by damaging chondrocytes. Lipocalin, in contrast, has anti-inflammatory properties and inhibits MMPs while suppressing inflammatory molecules such as IL-6 and TNF-α. VitD raises lipocalin levels while decreasing leptin levels, lowering inflammation and protecting against OA [129]. In addition, VitD may alleviate the progression of OA by regulating the ideal condition of the skeletal response to the disease’s pathological process. In a rat model of VitD deficiency, cartilage matrix degradation and proteoglycan loss occurred, which is comparable to early OA, demonstrating the detrimental effects of low VitD levels on cartilage [130]. The expression of MMP-9 and MMP-13 induced by TNF-α could be suppressed in vitro by vitamin D. VitD may have a cell-state-dependent effect on chondrocytes and could effectively regulate chondrocytes in the OA state [131]. VitD inhibits the beginning of mTORC1 complex-induced autophagy in chondrocytes by downregulating mTOR levels and then blocking the AMPK-mTOR signaling pathway. Increased autophagic flow in OA synovial membranes following VitD therapy protects against cartilage destruction. In OA animals, an additional VitD therapy reduced pain, cartilage damage, and inflammation, as well as caused a substantial drop in synovial MMP-13, IL-1, and MCP-1 levels [132]. By influencing the conversion of the macrophage polarization phenotype, VitD modulates the inflammatory response in OA. M2 appears mostly in the late phase of inflammation and has anti-inflammatory effects, and VitD promotes the preponderance of M2 phenotype macrophages in inflammation. VitD supplementation increases the expression of VDR and IL-37 in macrophages, reduces inflammation, and controls immunological function [133].

**Figure 4:**
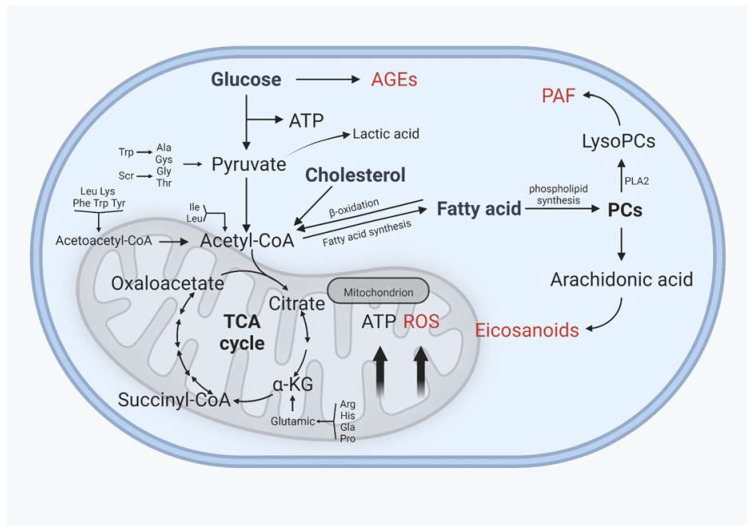
Interactions between glucose and protein metabolism and lipid metabolism in chondrocytes. AGEs: advanced glycation end products, ROS: reactive oxygen species, PAF: platelet-activating factor. AGEs, ROS, PAF, and eicosanoids are implicated in the inflammatory process of osteoarthritis, and their levels rise as OA progresses. (Figure was created with Biorender.com and accessed 20 September 2022.)

Lipid metabolism interacts closely with glucose metabolism and protein metabolism, not only in the traditional metabolic organs, but also in cartilage, in a relatively slower grade (Figure 4). Glucose is an essential source of energy, is involved in the composition of the extrachondral matrix, is engaged in signaling and metabolic control, and plays a crucial role in cartilage development and maintenance [134]. The transport of glucose, which requires the involvement of glucose transporters, is the first key step in glucose metabolism. Glucose transporter 1 (GLUT1), which regulates glucose transport, permits chondrocytes to continue developing normally. GLUT1 deficiency impairs cartilage development, whereas GLUT1 overexpression causes excessive glucose uptake and the production of advanced glycation end products (AGEs), resulting in cartilage degradation. After glucose is transported to the chondrocytes, it can either contribute to the synthesis of other substances or undergo glycolysis [135,136]. Under normal oxygen levels, one molecule of glucose enters glycolysis for metabolism, resulting in the production of two molecules of pyruvate, which then enter the mitochondria for oxidative phosphorylation. Pyruvate enters the tricarboxylic acid cycle via oxidative phosphorylation to produce 36 molecules of ATP, which serves as the cell’s energy source. In contrast, glucose is phosphorylated horizontally through anaerobic glycolytic substrates at low oxygen levels to generate a small amount of ATP, and eventually, lactate [137,138]. Because the oxygen supply decreases with depth in cartilage, articular chondrocytes have fewer mitochondria than other tissues, and ATP is mostly obtained by the Embden–Meyerhof glycolytic route, with only 10% of ATP derived from aerobic oxidation; however, mitochondrial activity is important in cartilage homeostasis and metabolism [139,140]. 

The tricarboxylic acid cycle is an important metabolic pathway that connects glucose, fats, and amino acids. Glucose is metabolized to produce acetyl coenzyme A, which can then be used to synthesize lipids, whereas glycerol in lipids is metabolized to produce glycerol phosphate, which can then be converted to glucose, and amino acids can be converted to glucose after deamination to produce alpha-keto acids, which can then be used as raw materials for phospholipids [141]. In OA cartilage, glucose and pyruvate consumption rose dramatically, as did the production of lactate and some TCA intermediates such as citrate, cis-aconitate, and malate, as well as glutamine and succinate levels. The tricarboxylic acid cycle acts as a connection between the three primary nutrients, and variations between distinct metabolic pathways may have consequences for cartilage homeostasis [142].

Different metabolic pathways are affected in pathological states, but the metabolic pathways are not isolated from one another; rather, they are intricately interconnected and serve distinct functions. Cholesterol accumulation in cartilage causes degenerative changes in cartilage. On the one hand, increased PLA2 activity and, thus, more PC conversion to AA activate eicosanoid and prostaglandin pathways [143]. On the other hand, n-6 PUFAs and SFAs act as proinflammatory substances, eventually leading to an inflammatory cartilage microenvironment. The ß-oxidation route catabolizes and provides energy from fatty acids, and carnitine plays an important regulatory function in mitochondrial fatty acid absorption. In OA, acylcarnitine levels are lowered and correspond with OA severity, reducing fatty acid catabolism and ATP generation [144]. 

Chondrocytes in OA exist in a proinflammatory milieu where hypoxia induces the elevation of pyruvate kinase M2 (PKM2) expression, reduces the energy supply through oxidative phosphorylation, and increases ATP generation by glycolysis. Chondrocyte proliferation and differentiation are hindered as a result of increased glycolysis [145]. Animal models of OA were developed using the mechanism through which monosodium iodoacetate (MIA) alters glycolysis by inhibiting glyceraldehyde-3-phosphate dehydrogenase [146]. Lactate dehydrogenase converts pyruvate to lactate during accelerated anaerobic glycolysis, creating a tiny quantity of ATP and causing lactate buildup and the creation of an acidic microenvironment. It has been demonstrated that creating an acidic milieu (the usual pH of cartilage is 6.6) inhibits chondrocyte extracellular matrix formation [147]. The greater ATP demand of chondrocytes in OA is related to increase NO generation. Arginine, a physiological nitrogenous substrate that targets iNOs, and NO generation in chondrocytes are linked to the pathophysiology of OA. Specific activation of arginase II in chondrocytes via the NF-κB pathway leads to the increased production of MMP-3 and MMP-13 in chondrocytes, resulting in cartilage degradation [148].

## 5. Lipid Metabolism and OA Treatment

Total plasma n-6 PUFAs and DHA are adversely related with cartilage degradation, whereas n-6 PUFAs are proinflammatory in the development of OA. Dietary lipid consumption influences systemic lipid levels, and the proper composition of dietary lipids may play an important role in preventing articular cartilage health. In an OA mouse model, adding Antarctic krill oil to the diet enhanced the intake of EPA-PL and DHA-PL and had a low n-6/n-3 PUFA ratio to block articular cartilage deterioration and slow the course of OA [149]. According to one study, dietary treatment with tiny levels of n-3 PUFAs helped repair damaging OA and decreased the consequences of obesity. Dietary SFAs and n-6 PUFAs, on the other hand, increase the development of OA, and food and metabolism appear to be more relevant than body weight in the relationship between obesity and post-traumatic OA [75]. Hypercholesterolemia causes mitochondrial dysfunction in chondrocytes, resulting in increased ROS and apoptosis, and antioxidants and statins have been shown in animal experiments to help preserve cartilage [149]. ABCA1 is essential for cholesterol metabolism. In OA, the expression of serum and glucocorticoid-inducible kinase 1 (SGK1) is increased, and ABCA1 exerts action mediated by the camp response element-binding protein (CREB1). As SGK1 silencing stimulates chondrocyte growth and reduces inflammation, it may be a unique method for treating OA [150]. In OA cartilage, the increased expression of miR-155 leads to the downregulation of cholesterol efflux-related gene expression such as LXR, resulting in cholesterol buildup and the development of inflammation, whereas the serine protease inhibitor vaspin (a protein released by white adipose tissue) has been found to inhibit miR-155 expression and enhance cholesterol efflux, therefore slowing the progression of OA [151]. The use of slow-release products containing PLA2 inhibitors can inhibit PLA2, hence lowering inflammatory reactions induced by membrane phospholipid hydrolysis that generates inflammatory mediators such as AA and lysophospholipids [152]. S1P has been demonstrated to inhibit IL-1-induced iNOS, ADAMTS-4, and MMP-13 expression [153]. MSC MVs require S1P for enrichment and penetrate cartilage in a cd44-dependent way to drive chondrocyte proliferation, matrix deposition, and cartilage defect repair, and S1P blockage inhibits the effect of MSC MVs on cartilage repair [154]. The investigation of S1P agonist-like medicines has the potential to lead to a significant advancement in the treatment of OA. The effects of lipid metabolism on cartilage are summarized in Table 1.

## 6. Conclusions

OA is a complicated disease with numerous risk factors, including trauma, age, obesity, genetics, and metabolism. The majority of the previous research on OA has focused on traumatic OA, but with the global increase in obesity rates, the role of obesity and metabolism in OA has also received increased attention. Obesity not only increases pressure load to the joints, but the hypercholesterolemia and inflammatory response caused by some adipokines associated with obesity can also cause cartilage damage [155]. Numerous alterations in lipid metabolism, including cartilage, subchondral bone, and periosteum, are involved in the pathogenesis of OA, and these alterations interact with inflammatory mediators to influence the development of the lesion. Previous research on the effect of lipids on cartilage has paved the way for the development of novel articular cartilage lesion treatments. Altering a specific lipid metabolism pathway with drugs or diet may be a potential treatment target for articular cartilage disease. Because the specific mechanisms underlying the pathogenesis of OA remain unknown, and there are only a few options for cartilage treatment, a metabolic approach could be an alternative. The exploration of lipid metabolism and clear metabolic pathways must be expanded, and further investigation of the sites of action of lipid metabolism on the function of cartilage and its homeostasis under normal physiological conditions is still required.

## Figures and Tables

**Table 1:** Summary of the effects of lipid metabolism on cartilage.

Author, Year	Study Population	Methods	Main Outcomes
Shufang Wu, 2003 [99]	Sprague Dawley rats	Treat with inhibitor of the cholesterol synthesis	Cholesterol production suppression inhibits chondrocyte proliferation, hypertrophy, and differentiation. Cholesterol controls growth plate chondrogenesis and longitudinal bone formation, potentially through enhancing IHH’s action in the growth plate.
H. Kishimoto, 2010 [104]	Bovine articular chondrocytes	Treat with ox-LDL	ox-LDL stimulates the hypertrophy of OA chondrocytes, in part, through binding to LOX-1. The binding of ox-LDL to LOX-1 promotes intracellular ROS generation and oxidative stress in chondrocytes.
Aspasia Tsezou, 2010 [106]	Human chondrocytes with OA	Treat with LXR agonist	In osteoarthritis patients, the expression of ABCA1 and ApoA1 transcriptional regulators LXRa and LXRb was dramatically reduced. LXR agonists enhance cholesterol efflux in chondrocytes with osteoarthritis.
C. Gentili, 2005 [109]	Chick chondrocytes	Treat with LXR/RXR agonists	Chondrocytes manufacture ApoA1 during development, and ligands that activate LXR or RXR may lead to a significant increase in ApoA1 expression and cholesterol efflux in thicker chondrocytes. Other LXR target genes are implicated in cholesterol transport and homeostasis, including ABCA1 and SREBP1. The expression of chondrocyte SAA is inhibited by ligands that activate LXR or RXR and induce ApoA1.
Dominique de Seny, 2015 [110]	Human chondrocytes with OA	Incubation with ApoA1, rhSAA, HDL, LDL	By boosting TLR4 activation, free ApoA1 particles in OA joints may directly or indirectly contribute to the local inflammatory process. ApoA1’s inflammatory characteristics on chondrocytes and fibroblast-like synoviocytes will be influenced by HDL and LDL concentrations that are tightly regulated.
Wouter de Munter, 2013 [112]	LDL receptor-deficient and wild-type control mice with OA	Cholesterol-rich diet	In OA, elevated LDL cholesterol levels stimulate the development of ectopic bone. The underlying process may include TGF -β activation and, to a lesser degree, BMP activation after ox-LDL absorption by synovial intimal macrophages.
W an-Su Choi, 2019 [113]	Mice with OA	High-cholesterol diet vs. regular diet	In osteoarthritic chondrocytes, increased cholesterol absorption, cholesterol hydroxylase levels, and hydroxysterol metabolite synthesis directly activate ROR, contributing to the pathogenesis of OA. Through LOX1-mediated augmentation of CH25H and CYP7B1 absorption and metabolism, increased cholesterol levels in chondrocytes induce experimental OA in mice. The CH25H-CYP7B1-ROR axis of cholesterol metabolism in chondrocytes is a crucial catabolic regulator in the etiology of osteoarthritis.
Margaret Man-Ger Sun, 2020 [114]	Mice primary chondrocytes	Treat with specific LXRagonist	Upon activation by its specific agonist, the LXR-Srebp1-Scd1 axis functions as a cholesterol sensor, lowering the buildup of free cholesterol in cells to mitigate cholesterol’s cytotoxic effects. LXR is activated by its particular agonist, and LXR’s protective impact on OA may be mediated, in part, by increased cholesterol excretion and suppression of chondrocyte hypertrophy.
Fotini Kostopoulou, 2015 [116]	Human cartilage with OA	Treat with TGF-β1	miR-33a may influence the TGF-1/PI3K/Akt signaling pathway via the regulation of Smad7 expression. MiR-33 inhibition in OA chondrocytes raises the levels of ABCA1 and HDL, resulting in a reduction in MMP-13 expression.
Shirou Tabe, 2017 [122]	ATDC5 cells	Silence LPCA4 expression	Other than Col2, Sox9, and Runx2, knockdown of LPCAT4 decreased the mRNA expression of chondrogenic differentiation-related molecules. The expression of LPCAT4 rises during chondrogenic differentiation, and LPCAT4 is implicated in chondrocyte hypertrophy.
Mi-Kyoung Kim, 2006 [124]	Rat primary chondrocytes	Treat with S1P and PhS1P	Sphingosine kinase-1 is the key to the production of S1P from sphingosine. S1P and PhS1P lead to cellular proliferation of rat primary chondrocytes through a ptx-sensitive G-protein-mediated pathway, plc-mediated [Ca^2+^] increase and activation of MAPKs.
Hongming Miao, 2015 [79]	Male C57BL/6 mice	HFD	In a way reliant on LDH-a, circulating stearic acid elevates lactate levels in plasma and chondrocytes. Stearic acid increases the production of VEGF and cytokines that cause inflammation through a TLR4 pathway and a new lactate/HIF1 pathway.
Oscar lvarez-Garcia, 2014 [80]	Human chondrocytes and fibroblast-like synoviocytes	Human chondrocytes from normal and OA were treated with Palmitate or oleate in combination with IL-1	Palmitate, but not oleate, stimulates IL-1 in normal chondrocytes, causing caspase activation and cell death, and upregulates IL-6 and COX2 in chondrocytes and fibroblast-like synoviocytes through Toll-like receptor-4 signaling.
Daniel edina-Luna, 2017 [81]	Human chondrocytes	Treatment with FFA blend consisting of palmitic and oleic acids vs. control	A high-fat milieu causes cartilage degradation by raising chondrocyte IL-1, ROS, and RNS, promoting autocrine production of IL-6 and IL-8.
Chia-Lung Wu, 2014 [75]	Mice with OA	HFD, rich in SFAs, n-3 PUFAs, n-6 PUFAs	n-3 PUFA supplementation may reduce the effects of obesity on OA and enhance healing. SFAs and omega-6 PUFAs are both harmful in OA following joint damage. SFAs may cause synovial macrophages to release IL-1 and TNF, both of which are implicated in cartilage degradation.
M. Miyamoto, 2003 [88]	Primary chondrocytes from rat rib cartilage	Treat with PGE2	Simultaneous stimulation of EP2 and EP4 induces chondrocyte differentiation
H. Davis Adkisson, 1991 [85]	Cartilage from multiple species	Argentation high-performance liquid chromatographic(AHPLC) separationof fatty acid	Low levels of n-6 PUFAs and high levels of abnormal n-9 fatty acids found in normal cartilage
Kohei Nishitani, 2010 [89]	Human chondrocytes	Treat with PGE2	PGE2 inhibits IL-1β-induced MMP-1 and MMP-13 synthesis through EP4 activation and suppression of MKK4, JNK MAP kinase, and c-JUN phosphorylation.
Sunderajhan Sekar, 2017 [73]	Rat	High-Carbohydrate Supplements with SFAs vs. cornstarch diet	High-fat diet can induce metabolic syndrome in rats. Palmitic acid and stearic acid combined with IL-1 mediate cartilage damage by increasing MMP-13, ADAMTS-4, and ADAMTS-5 gene expression and increase the severity of OA
Sujeong Park, 2022 [74]	ACOT12 knockout mice	Treatment with specific agonists and inhibitors of PPARα	ACOT12 is involved in maintaining cartilage homeostasis. Increased accumulation of acetyl-CoA due to PPARα deficiency leads to stimulation of cartilage-degrading enzymes and chondrocyte apoptosis through regulation of ACOT12.

## Data Availability

Not applicable.
